# Using a new video rating tool to crowd-source analysis of behavioural reaction to stimuli

**DOI:** 10.1007/s10071-021-01490-8

**Published:** 2021-03-09

**Authors:** Holly Root-Gutteridge, Louise P. Brown, Jemma Forman, Anna T. Korzeniowska, Julia Simner, David Reby

**Affiliations:** 1 Mammal Vocal Communication and Cognition Research Group, School of Psychology, University of Sussex, Brighton, BN1 9RH UK; 2 MULTISENSE Lab, School of Psychology, University of Sussex, Brighton, BN1 9RH UK; 3 Equipe Neuro-Ethologie Sensorielle, ENES, CRNL, CNRS UMR5292, INSERM UMR_S 1028, University of Lyon, Saint-Etienne, France; 4 School of Life Sciences, Joseph Banks Laboratories, University of Lincoln, Beevor Street, Lincoln, LN6 7DL UK

**Keywords:** Analysing behaviour, Animal behaviour metrics, Rating behaviour, Coding behaviour, Crowd-sourcing data analysis, Measuring behaviour

## Abstract

Quantifying the intensity of animals’ reaction to stimuli is notoriously difficult as classic unidimensional measures of responses such as latency or duration of looking can fail to capture the overall strength of behavioural responses. More holistic rating can be useful but have the inherent risks of subjective bias and lack of repeatability. Here, we explored whether crowdsourcing could be used to efficiently and reliably overcome these potential flaws. A total of 396 participants watched online videos of dogs reacting to auditory stimuli and provided 23,248 ratings of the strength of the dogs’ responses from zero (default) to 100 using an online survey form. We found that raters achieved very high inter-rater reliability across multiple datasets (although their responses were affected by their sex, age, and attitude towards animals) and that as few as 10 raters could be used to achieve a reliable result. A linear mixed model applied to PCA components of behaviours discovered that the dogs’ facial expressions and head orientation influenced the strength of behaviour ratings the most. Further linear mixed models showed that that strength of behaviour ratings was moderately correlated to the duration of dogs’ reactions but not to dogs’ reaction latency (from the stimulus onset). This suggests that observers’ ratings captured consistent dimensions of animals’ responses that are not fully represented by more classic unidimensional metrics. Finally, we report that overall participants strongly enjoyed the experience. Thus, we suggest that using crowdsourcing can offer a useful, repeatable tool to assess behavioural intensity in experimental or observational studies where unidimensional coding may miss nuance, or where coding multiple dimensions may be too time-consuming.

**Supplementary Information:**

The online version contains supplementary material available at 10.1007/s10071-021-01490-8.

## Introduction

Developing accurate and unbiased measures of behavioural responses to stimuli is critical to the study of animal behaviour (Banks 1982; Meagher 2009). The human brain remains one of the most effective tools for analysing data, encompassing a wide range of features and achieving complex and high-level perceptual categorisations in milliseconds (Marois and Ivanoff 2005). Observation can therefore be a powerful method for characterising animal behaviour. However, the use of subjective assessments of behaviour, personality, and emotional state has attracted strong criticism due to biases resulting from prior experience, preconceptions, and observer-gender (Marsh and Hanlon 2004; Tuyttens et al. 2014). Here we use the term subjective to describe metrics based on “an individual’s perception and judgement, and can therefore be influenced by experience or personal views” (Meagher 2009). Therefore, much effort has been expended on developing robust, discrete coding systems to quantify distinct behaviours. A common example of this is the *ethogram*, which is a list of species-specific behaviours coded into behavioural units which represent discrete actions, coded with duration, latency, and binary occurrence (Banks 1982). However, ethograms require the choice of behaviours to be made a priori, and can fail to capture subtle responses (e.g. small ear movements), or complex interactions of many different elements (e.g. the combination of ear, eye, and mouth movements) (Meagher 2009; Waller and Micheletta 2013). Objective assessments may also fail to fully describe the gestalt as it is perceived by the observer (Meagher 2009). Observers can identify emotional valence and arousal from gestalt impressions and may use fleeting or tiny movements to guide their observations (Tami and Gallagher 2009; Wan et al. 2012; Scheumann et al. 2014; Maréchal et al. 2017; Kelly et al. 2017; Guo et al. 2019). Moreover, the lack of blinding during analysis has proven to be problematic, especially when experimenters analyse their own work (Tuyttens et al. 2014). The problems associated with rating behaviours have resulted in the understandable perception that quantitative coding is more objective and reliable, and therefore superior, and coding is used to validate rating results (see Vazire et al. 2007 for a review). Here we explore whether observer bias can be reduced by simply increasing the number of observers (e.g. by using a crowdsourcing approach to recruit naïve observers), and whether this can result in reliable metrics of one feature of animal behaviour, i.e. reaction strength.

Although used only rarely by behavioural scientists, crowdsourcing has been used for over a century by biologists (Droege 2007). This has produced large-scale datasets which could not otherwise be generated (e.g. surveying bird species distributions at national or international levels by using local reports by ornithologists) (Desell et al. 2015). More recently with the expansion of the internet, researchers have adopted crowdsourcing approaches for mass data analysis tasks which are time-consuming or cannot be automated easily (Cox et al. 2015). These have been found to contribute positively to both public engagement levels and science generally, with high publication rates and large cost savings relative to employing a worker full-time for years (Cox et al. 2015). Canine behaviour studies have previously used crowdsourcing for both data collection (Stewart et al. 2015; Worsley and O’Hara 2018) and analysis (Mirkó et al. 2012; Bloom and Friedman 2013), and while this has not been widely adopted by researchers, it has shown that even naïve observers can form correct assessments of dog behavioural responses (Mirkó et al. 2012) and emotions (Wan et al. 2012; Bloom and Friedman 2013). Furthermore, humans can successfully judge a domestic dog’s emotions from its facial expressions in photographs, independent of their knowledge of dogs, suggesting that personal experience is not required (Bloom and Friedman 2013) and that naïve and inexperienced observers can still offer valuable analyses.

Crowdsourcing represents a feasible option for the mass-analysis of data despite trade-offs between the time-saved and the loss of expertise, especially as the importance of expertise depends on the ambiguity of the data being presented (Law et al. 2017). While individuals may make mistakes, errors are minimised by drawing data from many different people, with results collated and reviewed, so the advantages of crowd-sourced data analysis typically outweigh the errors (Bonter and Cooper 2012; Gardiner et al. 2012). The reliability of observers can be quantified using measures of correlation between individuals, referred to as inter-observer reliability, such as Cronbach’s alpha, and the measures accepted if the agreement is high enough (Bland and Altman 1997; Koo and Li 2016). Thus, we suggest that greater objectivity can be achieved by increasing the number of observers to reduce the influence of any single observer on the results and that as the observers are blind to the aim of the experiment, the potential subjectivity in ratings can be reduced as there is no bias towards desired outcomes.

In this study, we explored whether crowd-sourcing analysis by large groups of naïve participants can produce widely-agreed-upon assessments of the behaviour of dogs, and we also investigate how many observers are required to achieve a reliable result. We follow a model detailed by Hecht and Spicer Rice (2015), where researchers provide the data content which is to be assessed and naive observers analyse them. Observers were presented with videos of domestic dogs responding to acoustic playback trials and asked to rate the strength of the dog’s reaction to the stimulus in each video. Raters were not given instructions as to which behaviours should be considered, or other criteria for response, and were asked for their naïve judgements only. Finally, as crowdsourcing relies on the willingness of participants to perform the task, the participants were asked to rate their enjoyment of the study and the likelihood of participating again in future.

## Methods

### Participants

Three hundred and ninety-six people (56 men and 340 women) participated in the study, with a mean age of 19.92 years old, standard deviation (SD) = 4.0 years, and the oldest participant was 67. 16 people were known to have rated more than one set of videos. The participants were recruited by word of mouth and via the University of Sussex student body (contacted via online advertising on an internal website). Students were rewarded with course credit through the School of Psychology’s system while no reward was offered to other participants. The results for the studies were pooled. 358 (90.4%) participants agreed with the statement (1) “are you an animal lover?” and 199 participants (50.3%) agreed with statement (2) “Have you ever owned a dog?” Overall, the participants had owned dogs from 96 breeds and identified breed-mixes (see Electronic Supplementary Materials for list of breeds owned).

### Videos: dog behavioural reactions

Each rated video featured a single dog listening to a short stimulus sound, with all dogs recorded in the same location. All video data were collected as part of the BBSRC funded project ‘How Dogs Hear Us’. The dogs were accompanied by their owners to a testing room on the UoS Falmer campus where they each heard six stimuli sound in a habituation-dishabituation experiment [see Root-Gutteridge et al. (2019) for details]. None of the stimuli was distressing to the dogs.

Each video was clipped using the video editing software iMovie (Apple Inc., 2016) or Sony Vegas Pro (version 9: Sony Creative Software, 2009; version 13: Sony Creative Software, 2013; version 14: Sony Creative Software, 2014) to feature a single dog’s response to one trial. Soundtracks were muted, with the stimulus sounds replaced by a champagne cork pop sound effect (see electronic supplementary material (ESM) for sample video). This replacement was to avoid bias in the raters’ responses, which can be an issue in studies without blinding (Tuyttens et al. 2014). The videos were converted to MP4 format using Adobe Media Encoder CC (Adobe 2018).

A total of 258 videos were presented in three separate datasets (wave 1:36 videos, wave 2:78 videos, wave 3:144 videos, where wave indicates a dataset). These videos were selected to represent a range of behavioural reactions from low to high activity within the context of the experiment.

### Human ratings

Rater participants were logged on our in-house testing platform (www.syntoolkit.org), which allows video uploads alongside surveys, and questionnaires. The first task for participants was to fill in a short questionnaire about their age, gender, whether they had ever been a dog owner, and, if so, which breeds they had owned. Then participants were shown four sample videos of different dogs’ reactions to demonstrate the range of possible dog reactions from low activity (only the dog’s eyebrows moved) to high activity (the dog’s entire face and body moved). Participants were not asked to rate these videos and were not informed how these videos had been rated by the researchers, to avoid biasing their assessments, but were told that they represented the range of reactions.

**Fig. 1:**
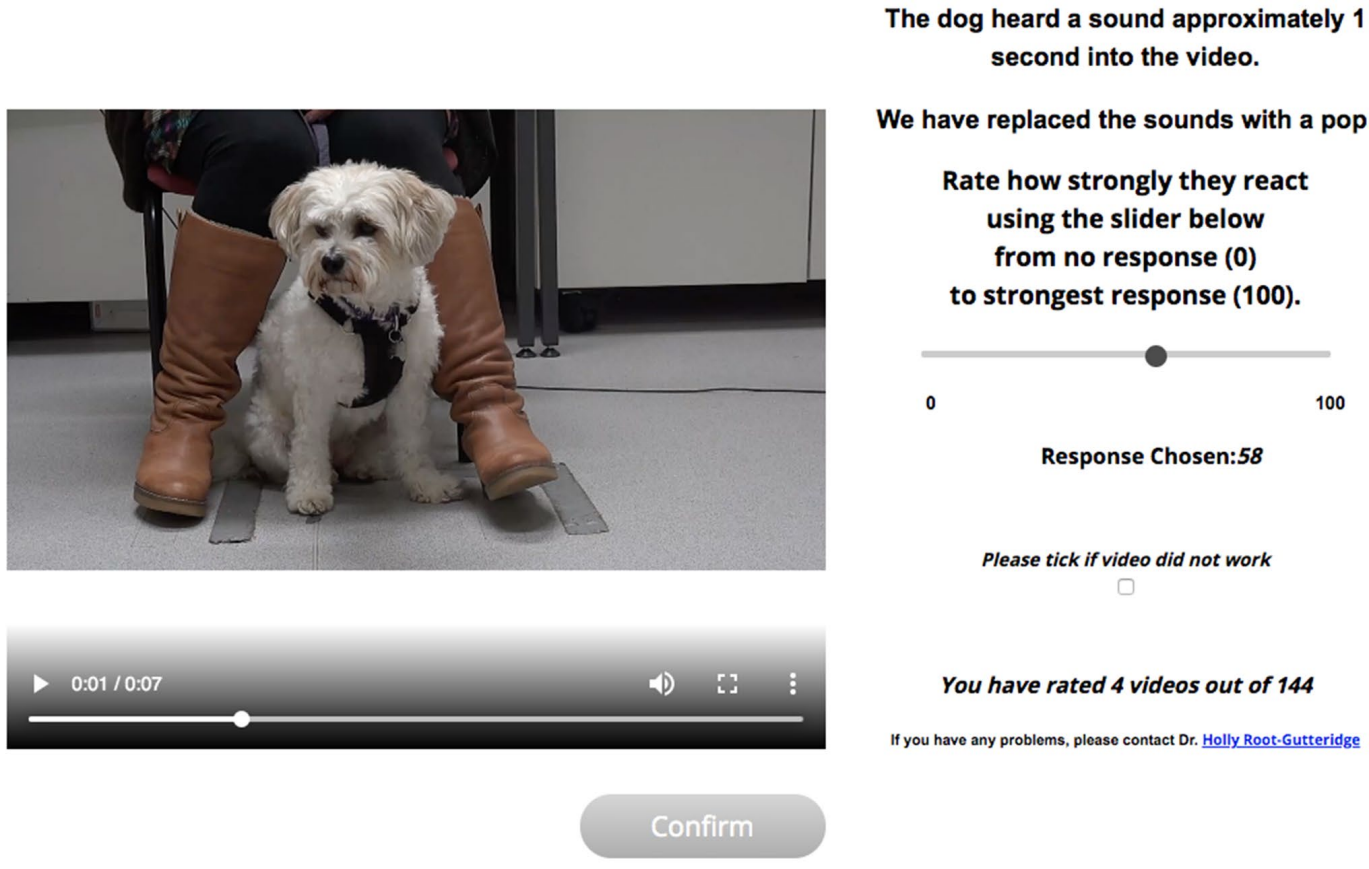
Example screen from the survey website for rating dogs’ responses. The video started when the participant clicked play. Dogs had heard the sound stimulus approximately 1 s into the video (see Electronic Supplementary Material for example video) but the sound was replaced within the playback video by a “pop”

Next, participants were asked to watch dog videos to rate the strength of the dogs’ reaction to stimuli using a slider bar running from 0 (no reaction) to 100 (strongest reaction), with the slider bar pre-set to zero to avoid priming the participants’ responses. Videos were presented with one per page (see Fig. 1). Participants were asked to watch each video before rating the dog’s reaction to the sound and could watch the video as many times as they chose. The website randomised the order of presentation and recorded the order of presentation along with the ratings. Videos only played when clicked and could be rewound or paused as desired. A tally displayed how many videos had been completed so far. Results were saved after each video by pressing the “Confirm” button and videos could not be viewed again once “Confirm” was selected.

### Video ratings: pilot study

We piloted the study and website with an initial presentation of 36 videos of 6 dogs, with 6 videos per dog. These were initially shown to 10 naïve participants. In addition to rating the dogs, participants were asked to rate the website’s instructions, ease of use, and presentation, and to comment on any issues they experienced. Following this, we adjusted the slider bar for ease-of-use, added additional instructions, and included a check-box to indicate if the video had not worked as expected.

### Video ratings: pooled results from waves 1–3

Following the pilot, the study was rolled out through the University of Sussex internal study recruitment website. 346 additional participants were recruited to rate three separate sets of videos, hereafter referred to as waves 1, 2 and 3. The pooled 216 participants rated a total of 258 videos: 29 participants, including the initial 10, rated the 36 videos of 6 dogs originally used in the pilot (wave 1), 153 participants rated 78 videos of 13 dogs (wave 2), and 34 participants rated 144 videos of 24 dogs (wave 3). The videos were chosen to represent a range of reactions by the dogs including those which moved only slightly and dogs which produced complex movements in response to the stimulus. One dog provided twelve videos presented across two waves, while all other dogs provided 6 videos to one wave. The videos in wave 3 represent all the videos recorded for one habituation-dishabituation test condition in Root-Gutteridge et al. (2019), which were not used in that study as too many of the dogs were distracted, looking at their owners, or did not reach habituation. Thus, the wave 3 videos represent a complete set of experimental videos.

### Post-study questionnaire

Several months after the completion of the ratings for waves 1 and 2, participants were sent an invitation to participate in a questionnaire on their experience of rating dog behaviour videos, hosted on the site Survey Monkey. For wave 3, this questionnaire was added to the SynToolkit website and integrated into the task. Seven questions were asked (supplementary Table 1) and results were collated.

### Statistics

All statistics were performed in the statistical program SPSS 25 (IBM Corp. Released, 2013).

### Assessing inter-rater-reliability and required numbers for representation

Rater reliability is a measurement of how well different raters agree with each other and measures the homogeneity of assessments by different observers. Low inter-rater reliability suggests that raters do not assess the data using the same criteria. The probability of their agreement is assessed using the SPSS function ‘Reliability Analysis’. This uses the intra-class correlation coefficient (ICC) calculated using a two-way mixed model (Koo and Li 2016). This results in a Cronbach’s alpha score for the rating reliability, where > 0.8 is considered to be highly reliable (Bland and Altman 1997). It was calculated for each of the three waves independently as different raters had participated in each wave.

Following this, we estimated the number of raters required to achieve a similar level of inter-rater agreement as found in this study, i.e. > 0.8 following (Bland and Altman 1997), to inform how many raters might be required for similar studies. The number of raters was estimated from r = 2/cv, where r = required number of raters, cv = standard error of percent agreement/percent agreement across each video (Gwet 2010).

### Statistics for rating results

All rating results were pooled. Participants were identified using anonymised codes, which linked to the results from the questionnaires to allow their demographic information to be added to statistical models. The rating results were then compared in a single mixed effects linear model with sex, age, and agreement with statements (1) “Are you an animal lover?” and (2) “Have you ever owned a dog? “ as fixed effects, with individual rater ID and dog ID as random effects.

### Assessing behaviours performed in videos

When all the videos had been rated by more than 30 participants, the behaviours the dogs presented in each video were first listed (Table 1) and then assessed as a binary response (e.g. head turn? Y/N) for each video. Behaviours from all videos were coded independently by three researchers (co-authors JF, LB, and HRG). Where the agreement was not reached unanimously, the majority vote ruled.

**Table 1:** Description of behaviours performed by dogs in videos in response to stimuli and percent of videos where dogs performed the behaviours

Behaviour	Description	Videos where behaviour was performed (%)
Change in breathing	Dog showed altered breathing	8.1
Down^a^	Dog lay down from sit or stand	4.7
Ears moved	Dog changed ear position	50.0
Eyebrow movement	Dog moved its eyebrows	54.7
Eyes turned	Dog moved eyes independent of head movement	56.2
Facial expression	Dog changed facial expression	16.7
Freeze^a^	Dog stopped any movement	1.9
Head tilt	Dog tilted its head from centre to left or right	17.1
Head turn	Dog moved its head in the direction of the speaker	58.5
Look at speaker	Dog looked towards the speaker	54.4
Mouth moved	Dog opened or closed mouth	14.0
Nostrils flared	Dog flared nostrils	11.2
Retreat^a^	Dog moved away from speaker	0.8
Sit^a^	Dog moved to sit from down or stand	1.9
Stand^a^	Dog stood up from sit or down	3.9

^a^These measurements were removed from further analyses as they were observed in < 5% of videos

A principal components analysis (PCA) was performed to reduce the listed behaviours to a set of components (varimax rotation) in SPSS. These were loaded with behaviours that were highly correlated to strength as rated by participants. A linear regression model was then used to assess which components contributed to the reaction strength-ratings, Bonferroni adjusted *p* = 0.0125.

For each video, the latency and duration of the dog’s overall response were coded by the authors. Attention to the stimulus was defined as the dog performing one or more active behaviours (see Table 1). Latency was defined as the time between the stimulus onset and the start of the dog’s reaction. The duration was defined as the start of any of the behaviours and the time that the dog stopped visibly responding, or the beginning of the next trial. Therefore, duration was capped at 7 s as this was the sum of the duration of the original stimulus sound and the six-second habituation time. Lack of response was coded as duration equals zero. We used linear regression to explore the potential correlation between reaction strength and the duration or latency of response (adjusted *p* = 0.025).

Finally, an ordinal scale of behavioural responses was created, and each video was given a score from 0 to 4 by HRG, with 10% second coded by ATK. The operational definitions are given in Table 2 with 0 equalling the weakest reaction (e.g. no visible change in expression, demeanour, body posture etc.) and 4 equalling the strongest. We used linear regression to explore the potential correlation between the ordinal score of response and reaction strength.

**Table 2:** Definitions for ordinal scores of dogs’ strength of reaction in response to stimuli

Ordinal scale	Response is seen as change in				
	Eyes/ears orientation	Breathing	Facial expression	Head position	Body posture
0	N	N	N	N	N
1	Y	N or Slight	N or Slight	N	N
2	Y	Y	Y	Slow or slight	N
3	Y	Y	Y	Fast or large	N
4	Y	Y	Y	Y	Y

## Results

### Participants

For wave 1, participants rated between 1 and 36 out of 36 total videos (mean = 34.2, SD = 7.2). For wave 2, participants rated between 2 and 78 out of 78 total videos (mean = 72.4, SD = 18.1). For wave 3, participants rated between 10 and 144 out of 144 total videos (mean = 136.3, SD = 27.7). To remove unrepresentative outliers, which were often due in wave 1 to the programme not playing the video and raters thus giving inaccurate scores of “0” because they could not play the video, rating scores were retained if they were within 2 SD of the mean for the stimulus video, calculated in SPSS using the ‘Descriptives’ function.

### Assessing inter-rater-reliability and required numbers

Within each wave, there was a strong average agreement on ratings as assessed using the ICC metric (Cronbach’s alpha): wave 1 ICC = 0.785, wave 2 = 0.949, and wave 3 ICC = 0.992. When we removed ratings that fell outside of two SD from the mean, to account for videos where the video failed and the rating defaulted to zero, agreement increased for all studies: wave 1 ICC = 0.990, wave 2 ICC = 0.995, and wave 3 ICC = 0.998.

The estimated number of raters required to reach a similar level of agreement differed across studies: for wave 1 required *n* = 16 raters, for wave 2 = 17, and wave 3 = 10, depending on the standard error of the original ratings.

### Participant characteristics affecting rating strength

The linear mixed effects model of all ratings showed that the participants’ ratings of dogs’ reaction strength were affected by participants’ sex (F_1,3762_ = 60.153, *p* < 0.001), with men giving higher average ratings than women, age (F_1,3766_ = 7.646, *p* < 0.001), and age (F_15,3765_ = 7.646, *p* < 0.001), and whether they agreed with the statement that they were “animal lovers” (F_1,3801_ = 37.084, *p* < 0.001), with disagreeing participants giving higher ratings. However, as participants were recruited using a School of Psychology internal system at the University of Sussex, 97% of participants were under the age of 25, 85.4% of the raters were female and 90.4% of participants agreed they were “animal lovers”, making the result for those variables potentially unreliable. There was no effect of the rater’s previous experience of dog ownership (F_1,3637_ = 1.127, *p* = 0.289, 52% were dog owners).

### PCA results

The Principal Component Analysis (PCA) was used to reduce the dimensions of the behavioural descriptive variables listed in Table 1 to a smaller set. PCA achieves this by converting observations of possibly correlated variables (e.g. “head turn” and “look at speaker” or “eyes turned” and “eyebrows moved”) to a set of uncorrelated variables called principal components which explain the largest variance in the data. The correlation matrix for these are shown in Table 3. Components with Eigenvalues of >  = 1 were retained. Thus, the first four components were retained and explained 68.2% of the variance. Components 1–4 explained 25.9%, 16.8%, 15.2%, and 10.4% of the data variance respectively.

**Table 3:** Rotated component matrix from PCA analysis. Loadings > 0.5 are marked in bold. 68.2% of data variance was explained

Variable	Component			
	Facial expression	Ears and eyes	Orientation	Head tilt
Change in breathing	**0.814**	0.071	− 0.177	0.08
Ears moved	0.147	**0.633**	0.363	− 0.142
Eyes turned	0.06	**0.746**	0.219	− 0.171
Eyebrows moved	0.031	**0.792**	− 0.283	0.062
Facial expression changed	**0.87**	0.095	0.142	0.005
Head tilt	− 0.083	− 0.252	0.046	**0.699**
Head turn	0.072	0.173	**0.834**	− 0.079
Looked at speaker	0.048	− 0.036	**0.788**	0.204
Mouth moved	**0.845**	0.041	0.21	− 0.07
Nostrils flared	0.095	0.054	0.044	**0.819**

### PCA components correlated with ratings reaction strength: LMM results

In this section we investigated what behaviours performed by the dogs were predictors of the reaction strength ratings. The linear regression model of the effect of observed behaviours on dog reaction strength determined that two PCA components had a significant effect on rating strength (Table 4), adjusted R-squared = 0.205 and F_4,253_ = 17.6. These were *Facial expression* (loaded with the behaviours Change in breathing, Facial expression changed, and Mouth moved) and *Orientation* (loaded with the behaviours Head turn and Looked at the speaker).

**Table 4:** Linear model results for PCA components of behaviours on rating scores

Fixed effect	Estimate	Std error	d.f	*t* value	*p* value
Facial expression	1.7244	0.7236	257	2.383	**0.018**
Ears and eyes	− 1.0675	0.7564	256	− 1.411	0.159
Orientation	5.8869	0.7824	254	7.524	** < 0.001**
Head tilt	1.0498	0.7008	257	1.498	0.136

Significant results are marked in bold

### Comparing reaction strength to classic metrics and ordinal scores

**Fig. 2:**
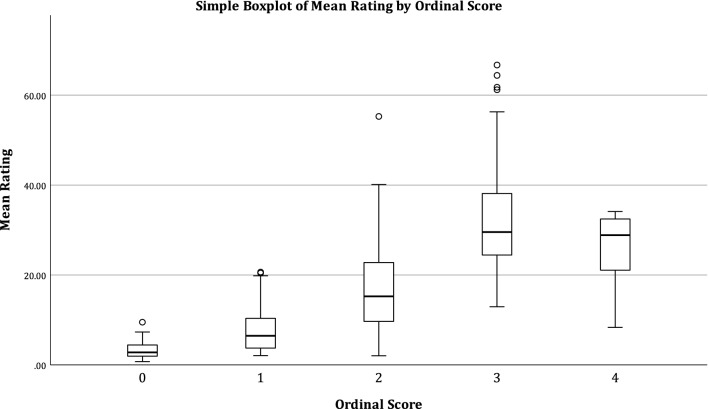
Boxplot of the mean rating of each of the 258 videos against the ordinal score of the intensity of reaction. Linear regression showed a correlation of score to rating at *r* = 0.716, *p* < 0.001

The linear regression of reaction strength to duration and latency of reactions was calculated for each wave and all waves together (Table 5). The duration was correlated to mean rating strength at *p* < 0.05 for all waves, though not at adjusted *p* value < 0.025 for waves 1 and 2, while latency was not correlated with reaction strength in any wave. However, latency was heavily skewed to the first 0.5 s after the stimulus began which meant it had limited variance. The linear regression of reaction strength was also calculated for ordinal scores. This was significant at *p* < 0.001 for all 3 waves and all waves together (Table 5, Fig. 2).

**Table 5:** Results for linear regression between mean rating strength and duration or latency for each wave and all results together, and for mean rating strength and ordinal score

Variable	Wave	*N*	*r*	*p*
Duration	1	36	0.332	**0.048**
	2	78	0.229	**0.044**
	3	144	0.430	** < 0.001**
	Pooled 1–3	258	0.389	** < 0.001**
Latency	1	36	− 0.019	0.913
	2	78	− 0.127	0.279
	3	144	− 0.065	0.456
	Pooled 1–3	258	− 0.115	0.073
Ordinal score	1	36	0.795	** < 0.001**
	2	78	0.712	** < 0.001**
	3	144	0.719	** < 0.001**
	Pooled 1–3	258	0.716	** < 0.001**

### Questionnaire results

107 participants returned the post-hoc survey questionnaire. Their results were collated and used to inform future directions (see Electronic Supplementary Material Table 1). 68.3% of people enjoyed the experience a moderate amount to a great deal (only 4% did not enjoy at all), while 82.3% agreed that they would participate in the same type of study again. 90.6% found the study easy to complete, and 92.5% felt it was appropriate for scientists to use citizen-science recruits for data analysis. The most frequent comments were for fewer videos and a greater range of dogs.

## Discussion

We found that crowdsourced observations of animal behaviour could produce consistent ratings of response strength, with the high agreement between raters across more than 258 videos. As raters were not aware of the scores being given by others, the independence of their scores and the consistency between scores suggest that reliable metrics were achieved. Rated reaction strength related to several different behavioural responses in dogs, including head, eye, and mouth movements. As the ten binary variables describing the dogs’ behaviour in the video explained only 68.2% of the data variance in the Principal Component analysis, we determined that the dogs’ responses were not fully described by them, however, as binary coding of the presence of different behaviours such as head-turning and ear movement only captured 68.2% of the data variance and rating strength did not correlate strongly to either duration of or latency to reactions. Furthermore, the ordinal scores correlated well with the average ratings (*r* = 0.716, *p* < 0.001), but did not capture the full variability of the data. While the scores worked well for the lower intensity reactions, more complex behaviours were harder to capture using simple ordinal scores. Therefore, we suggest that ratings could potentially capture nuance and encompass a gestalt that is not captured by classic metrics alone, supporting Meagher (2009), and that crowd-sourcing ratings could offer a potentially powerful tool for analysing animal behaviour. However, classic metrics can be used to validate ratings against a widely accepted standard method of analysing behaviour.

We also determined that inter-rater agreement was high enough to overcome potential subjectivity by individual raters. Inter-rater agreement was high with a result of Cronbach’s alpha > 0.9 for most comparisons, well above the suggested threshold for excellent reliability of > 0.8 (Bland and Altman 1997). We estimated how many individuals raters are required to produce representative results from the standard error of the mean, which resulted in a range from 10 to 17. This is much smaller than the number who participated, which were between 34 and 216 raters per wave, and suggests that fewer than 20 people are required per video for reliable assessments. The higher number may also have reflected technical difficulties that the first two waves experienced where zeros were recorded when videos failed to work properly. However, we believe that the number of raters required to achieve consistency would be easy to achieve.

We suggest that crowdsourcing offers a useful metric for rating animal behaviour that is holistic and less liable to subjective bias than ratings done by one or two observers, and may require only a small group of individuals. There are several advantages to rating through crowdsourcing, as while there was an initial time investment in the preparation of the files and the removal of the soundtrack, neither recruitment nor post-hoc analysis of results were time consuming compared to the large amount of data provided by the raters. Also, while individual results are indeed subjective, we find that the collective response has a high level of agreement and that this could potentially be obtained with as few as 10 raters. These results are in line with previous studies where observers formed correct assessments of dogs’ behavioural responses (Mirkó et al. 2012) and emotions (Wan et al. 2012; Bloom and Friedman 2013). This also did not require expertise in dog behaviour on the part of the raters as dog ownership did not predict ratings. While this new method still requires validation against a range of established behavioural and physiological variables as well as testing its performance in different experimental set-ups and in other species before crowd-sourced rating is generalised, we believe that we have shown here that the raters can come to robust agreements about simple metrics of attention and that these provide reliable and useful metrics. In future, the validity of this method will rely on the question being posed correctly and metrics properly applied, (e.g. asking “how strongly does the dog react?” vs “the dog reacts strongly, what is your rating of their intensity?”) but high levels of agreement across multiple raters and correlation with more classically accepted methods suggest that ratings are a powerful tool.

Crowdsourcing relies on the goodwill participation of the crowd and is only sustainable if sufficient interest in it can be generated. Therefore, we asked our participants how they felt about the study in a post-hoc survey. Most participants had a positive attitude to the experience and expressed willingness to participate in similar studies in future. While this should be explored with a larger sample of the general population, the popularity of citizen science sites such as Zooniverse.org and Dognition.com suggest that it is possible to recruit large numbers of people for such studies and therefore that our method is repeatable. We suggest it is likely that there is a general willingness to participate in such behavioural research and that it can engage the public interest, as for other crowd sourced science projects (Desell et al. 2015; Law et al. 2017). As our participants were mostly undergraduate psychology students, there may have been a bias towards participants who were potentially more painstaking than average in their responses as they were being rewarded with course credit. However, previous research has shown that crowdsourcing can be a reliable method of data analysis (review: Bonney et al. 2014), and future studies could incorporate a greater range of participants to test the effects of demographics, including age, educational level, and experience.

A clear next step for this methodology is testing it with a broader range of experimental data, including more complex or difficult to rate behaviours, and testing it with other species. Here, dogs were used as the focal species and the results may reflect raters’ familiarity with the species compared to other, less familiar animals. Half of our participants currently or had previously owned dogs, however, this had no effect on their ratings of behaviour, suggesting that prior experience of living with the focal species was not important. Animal lovers did score the reactions lower than non-animal lovers, which may reflect that the degree of interest in animal behaviour influences ratings. Thus, exploring ratings of the behaviour of a diverse range of species performing a range of different behaviours and a broader cross-section of participants could determine the usefulness of crowdsourced ratings. In particular, we suggest that studies investigating the effectiveness of crowd sourcing ratings of attention and emotional valence would be particularly valuable as these are notoriously difficult to categorise using classic methods.

In conclusion, we suggest that crowdsourcing can offer reliable assessments of the strength of response to stimuli and that it is a useful tool which could also benefit other behavioural observation studies.

## Supplementary Information

Below is the link to the electronic supplementary material.

Supplementary file1 (DOCX 24 KB)

## Data Availability

The videos are available via the Dryad depository under the title ‘Using a new video rating tool to crowd source analysis of the behavioural reaction to stimuli’: https://doi.org/10.5061/dryad.rbnzs7h95
