# Supplementary material for: Using a new video rating tool to crowd-source analysis of behavioural reaction to stimuli
Source: Anim Cogn. 2021 Mar 9;24(5):947–56. doi: 10.1007/s10071-021-01490-8 (PMC8360862; doi:10.1007/s10071-021-01490-8)
Supplement: Supplementary file 1 — Supplementary file1 (DOCX 24 KB) [file 10071_2021_1490_MOESM1_ESM.docx]

**Electronic Supplementary Material**

**Table 1 Results from the survey questionnaire administered after the rating survey was completed.**

| **Question** | **Answer options** | **Reply N** | **Percent** |
| --- | --- | --- | --- |
| 1. How many dog videos did you rate as part of the study? | <10 >10 <50  >50 | 14  43  27 | 16.7 |
|  |  |  | 51.2 |
|  |  |  | 32.1 |
| 1. Did you enjoy your experience of rating dog videos? | Not at all  A little  A moderate amount  A lot  A great deal | 4  30  35  22  16 | 3.7 |
|  |  |  | 28.0 |
|  |  |  | 32.7 |
|  |  |  | 20.6 |
|  |  |  | 15.0 |
| 1. Would you participate in this type of study again? | Strongly disagree  Disagree  Neither agree nor disagree  Agree  Strongly agree | 3  2  14  66  22 | 2.8 |
|  |  |  | 1.9 |
|  |  |  | 13.1 |
|  |  |  | 61.7 |
|  |  |  | 20.6 |
| 1. Would you recommend this type of study to others? | Strongly disagree  Disagree  Neither agree nor disagree  Agree  Strongly agree | 1  2  17  69  18 | 0.9 |
|  |  |  | 1.9 |
|  |  |  | 15.9 |
|  |  |  | 64.5 |
|  |  |  | 16.8 |
| 1. Was the study easy to complete? | Strongly disagree  Disagree  Neither agree nor disagree  Agree  Strongly agree | 1  7  2  58  39 | 0.9 |
|  |  |  | 6.5 |
|  |  |  | 1.9 |
|  |  |  | 54.2 |
|  |  |  | 36.4 |
| 1. Do you think it’s appropriate for scientists to invite members of the public to give some of their time to help with their research? | Strongly disagree  Disagree  Neither agree nor disagree  Agree  Strongly agree | 2  2  4  46  53 | 1.9 |
|  |  |  | 1.9 |
|  |  |  | 3.7 |
|  |  |  | 43.0 |
|  |  |  | 49.5 |
| 1. Was there anything about the study you would improve? | *Free text* | *E.g.*  *Fewer videos* | |
